# Role of the Neuropeptide S System in Emotionality, Stress Responsiveness and Addiction-Like Behaviours in Rodents: Relevance to Stress-Related Disorders

**DOI:** 10.3390/ph14080780

**Published:** 2021-08-08

**Authors:** Ann-Marie Tobinski, Virginie Rappeneau

**Affiliations:** Department of Behavioural Biology, University of Osnabrück, 49 076 Osnabrück, Germany; atobinski@uni-osnabrueck.de

**Keywords:** neuropeptide S (NPS), NPS receptor 1 (NPSR1), single nucleotide polymorphism, emotion, stress, addiction, behaviour, rodent

## Abstract

The neuropeptide S (NPS) and its receptor (NPSR1) have been extensively studied over the last two decades for their roles in locomotion, arousal/wakefulness and anxiety-related and fear-related behaviours in rodents. However, the possible implications of the NPS/NPSR1 system, especially those of the single nucleotide polymorphism (SNP) rs324981, in stress-related disorders and substance abuse in humans remain unclear. This is possibly due to the fact that preclinical and clinical research studies have remained separated, and a comprehensive description of the role of the NPS/NPSR1 system in stress-relevant and reward-relevant endpoints in humans and rodents is lacking. In this review, we describe the role of the NPS/NPSR1 system in emotionality, stress responsiveness and addiction-like behaviour in rodents. We also summarize the alterations in the NPS/NPSR1 system in individuals with stress-related disorders, as well as the impact of the SNP rs324981 on emotion, stress responses and neural activation in healthy individuals. Moreover, we discuss the therapeutic potential and possible caveats of targeting the NPS/NPSR1 system for the treatment of stress-related disorders. The primary goal of this review is to highlight the importance of studying some rodent behavioural readouts modulated by the NPS/NPSR1 system and relevant to stress-related disorders.

## 1. Introduction

The neuropeptide S (NPS) was originally described in 2004 [1] as the endogenous ligand for the orphan Gq-protein and Gs-protein coupled receptor GPR154 or GPRA, now renamed the neuropeptide S receptor 1 (NPSR1) [2,3,4,5]. The gene coding for the NPS precursor peptide is found in all tetrapodes except for fish and has a strong sequence conservation across mammalian species [6].

**Figure 1:**
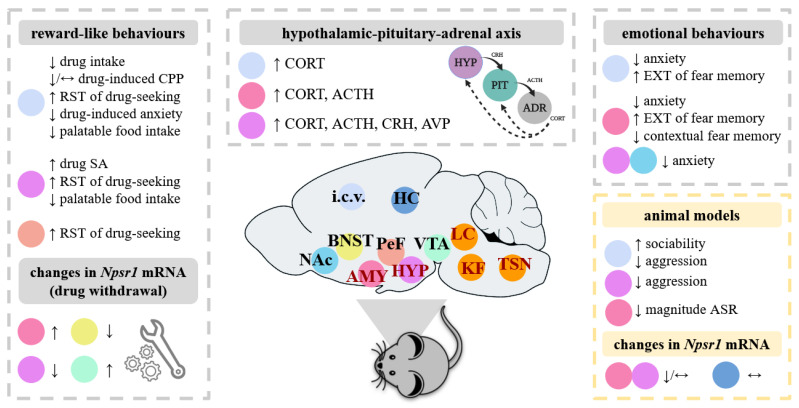
**Overview of the impact of the administration of NPS in various brain areas in rodents.** As depicted on this rodent brain (in dark red), the *Nps* precursor mRNA is expressed discretely in a few brain areas, including the trigeminal sensory nucleus (TSN), the locus coeruleus area (LC), the Kölliker–Füse nucleus of the lateral parabrachial area (KF), the hypothalamus (HYP) and the amygdala (AMY). By contrast to the *Nps* precursor mRNA, the *Npsr1* mRNA is widely expressed in the rodent brain, here shown in the AMY, the bed nucleus of stria terminalis (BNST), the hippocampus (HC), the HYP, the perifornical area (PeF), the nucleus accumbens (NAc) and the ventral tegmental area (VTA). Rodent models allow experimental investigation into the role of the NPS/NPSR1 system in addiction-like and emotional behaviours, as well as neuroendocrine and behavioural stress responsiveness. In the papers analysed in the present review (see summary in Tables 1 and 3), NPS was administered in various brain areas and the effects of NPS were observed at the behavioural, endocrine and molecular level in naïve animals. Similar effects of NPS were generally shown in rodents exposed to stress or with innate pathological phenotypes (here referred to as “animal models”), but other behaviours were affected and highlighted here. Abbreviations: ACTH, adrenocorticotropic hormone; ADR, adrenal; ASR, acoustic startle response; AVP, arginine vasopressin; CPP, conditioned place preference; CORT, corticosterone; CRH, corticotropin-releasing hormone; EXT, extinction; i.c.v., intracerebroventricular; NPSR1, neuropeptide S receptor 1; PIT, pituitary; SA; self-administration; RST, reinstatement. Symbols: ↑, increase; ↓, decrease; ↔, no significant change.

The NPS/NPSR1 system is expressed at the highest level in the brain, thyroid, salivary and mammary glands [1]. In the rat brain, the expression of the *Nps* precursor gene is limited to the trigeminal principal sensory nucleus, the lateral parabrachial nucleus, the peri-locus coeruleus area, the pontine central gray matter and few scattered neurons of the amygdala (AMY) and the hypothalamic dorsomedial nucleus [1,7,8]. In mice, the expression of the *Nps* precursor gene is restricted to the peri-locus coeruleus area and the Kölliker–Fuse nucleus of the lateral parabrachial nucleus area [9,10] (Figure 1). In humans, the *Nps* mRNA expression has only been examined in the pons and was found in the extension of the medial and lateral parabrachial nuclei, in the Kölliker–Fuse nucleus and around the adjacent lateral lemniscus and the pontine central gray matter [8]. 

In rodents, the *Nps* precursor mRNA co-localizes with those of galanin, vesicular glutamate transporter, choline acetyltransferase and corticotropin-releasing hormone, indicating possible interactions of NPS with associated neuromodulator and neurotransmitter systems [1,7,10]. A dense orexinergic fiber network has also been described to surround NPS-producing cells in the peri-locus coeruleus area, suggesting that orexin neurons may form synaptic contacts with NPS-producing neurons [10]. In contrast to the *Nps* precursor mRNA, the *Npsr1* mRNA is widely expressed in the rodent brain, mainly in the cortex, thalamus, hypothalamus (HYP) and in the AMY [1,7,9,11] (Figure 1). In terms of what has currently been examined in the human brain, the *Npsr1* mRNA is mainly located in the rostral laterodorsal tegmental nucleus, the cuneiform nucleus, the microcellular tegmental nucleus region and the periacqueducal gray [8].

The NPS/NPSR1 system was initially recognized as regulator of locomotion, arousal/wakefulness and fear and anxiety because of the pattern of *Nps* and *Npsr1* mRNA cerebral expression and owing to pioneering pharmacological and behavioural rodent studies [1,12,13,14]. Despite a large body of literature demonstrating the unique spectrum of NPS’s biological effects [15,16,17,18], the precise cellular and molecular mechanisms by which the NPS/NPSR1 system modulates such behavioural phenotypes are still unknown. Interestingly, some behaviours modulated by NPS are affected in stress-related disorders (e.g., post-traumatic stress disorder (PTSD), major depressive disorder (MDD) and anxiety disorders) [19]. Moreover, a potential association of the NPS/NPSR1 system with pathophysiological features of stress-related and substance use disorders has recently been highlighted [18,20]. Thus, the NPS/NPSR1 system may contribute to stress responsiveness and downstream stress-related phenotypes relevant to mental health.

Here, we conducted a narrative literature review in order to provide a comprehensive overview of the role of the NPS and its receptor in stress-related disorders and substance abuse.

## 2. The NPS/NPSR1 System and Stress-Relevant Endpoints in Rodents

The role of the NPS/NPSR1 system in stress-relevant behaviours and responses in rodents is summarized in Table 1.

### 2.1. Role of the NPS/NPSR1 System in Anxiety- and Fear-Related Behaviours

Following the pioneering study of Xu and colleagues that highlighted arousal and anxiolytic properties of NPS in mice, a wealth of pharmacological studies has reproduced such findings by using various behavioural paradigms based on approach-avoidance conflict and defensive behaviours in both male and female mice and rats ([21,22,23,24,25,26,27,28,29,30,31] but see [32,33,34]; see summary in Table 1). While the locomotor effects of central NPS infusion appeared mediated by corticotropin-releasing hormone receptor 1 (CRHR1), the anxiolytic-like effects of NPS were not [24]. 

The knockout of the NPS precursor peptide produced significant deficits in exploration and increased anxiety-related behaviour in mice [35], whereas the knockout of the NPSR1 had no major impact on locomotion and anxiety-related behaviour [26,30,33,36,37,38]. Results depended, however, on the behavioural test (i.e., anxiogenic conditions) as well as the mouse background strain (see summary in Table 2). Concerning fear-related behaviours, NPSR1 −/− mice showed no significant alterations in cued, contextual and social fear expression [36,37] or in safety learning [38]. The knockout of NPSR1 did not affect cued fear extinction but facilitated social fear extinction [36,39], as well as contextual fear extinction [37], and produced mixed findings on the acoustic startle response [37,38].

The infusion of NPS into the lateral and basolateral amygdala (LA/BLA) or into the medial amygdala (MeA) significantly reduced anxiety-related behaviour [40,41]. Similar anxiolytic-like effects were achieved using the adeno-associated virus-mediated overexpression of NPS into the MeA [42] or the infusion of NPS into the ventromedial hypothalamus, the paraventricular nucleus of the hypothalamus (PVN) and the ventral hippocampus [21,43,44]. The anxiolytic-like effects of intra-LA/BLA NPS were associated with enhanced glutamatergic transmission in mpara interneurons, possibly via NPSR1 in LA principal neurons [41], and they were antagonized by the NPSR1 antagonist SHA68 and an oxytocin receptor antagonist [21,43], indicating that the modulation of the behavioural anxiety response by NPS likely involves the oxytocinergic system. 

The central or the intra-LA/BLA infusion of NPS also facilitated the extinction of cued fear memory and reduced the contextual fear renewal [34,41], possibly reflecting the beneficial effects of NPS on memory [23,35,45,46]. Contrasting effects were obtained with the NPSR1 antagonist SHA68 [41]. Importantly, mice carrying the human-specific hypo-functional variant of the NPSR1 (referred to as the NPSR1-N107 mouse line for the non-synonymous SNP rs324981) displayed improved extinction of conditioned fear. This behaviour was mimicked by the pharmacological antagonism of the NPSR1 in the anterior basal amygdala of mice carrying the ancestral/wild-type NPSR1-I107 variant (NPSR1-I107 mouse line) [47] and thus warrants further investigation of the emotional phenotypes and anatomical correlates of the “humanized” NPSR1-I107N mouse lines.

### 2.2. Role of the NPS/NPSR1 System in Social Behaviours and Aggression

The NPS/NPSR1 system seems to have a minor impact on social behaviours and aggression in rodents, as indicated by the lack of effects of both NPS and the NPSR1 antagonist (d-Cys(tBu)5)NPS on sociability and social recognition [23,34,48] and the discrepant findings on aggression [48,49] in male rodents (see summary in Table 1). In line with the observations, no significant alterations in sociability and social novelty behaviours were found in NPSR1 −/− mice [39], although a slight increased innate level of aggression was revealed in male NPSR1 −/− mice [49] (see summary in Table 2).

### 2.3. Role of the NPS/NPSR1 System in Stress Responsiveness and Stress-Coping Behaviour 

The NPS/NPSR1 system appears to modulate the neuroendocrine stress response in male and female rodents. For example, NPS significantly increased plasma adrenocorticotropic hormone (ACTH) and corticosterone (CORT) level [30,50,51]. The temporal dynamics in the CORT rise induced by NPS infusion indicates that NPS directly acts on the PVN in order to regulate the hypothalamic-pituitary-adrenal (HPA) axis function. Importantly, the application of NPS to hypothalamic explants increased corticotropin-releasing-hormone (CRH) and arginine vasopressin (AVP), while the application of NPS to adrenal explants failed to stimulate ACTH release, indicating the direct effects of NPS on the HYP [50]. It is important to mention, however, that mice carrying the human-specific variant NPSR1-N107 showed no significant differences in the plasma CORT level compared to mice carrying the NPSR1-I107 ancestral variant, both under basal conditions and during fear memory retrieval [47]. Thus, further work is needed in order to understand how the NPS/NPSR system plays a role in activating the stress system.

Regarding behavioural stress responsiveness, the central infusion of NPS has been found to reduce the stress-induced hyperthermia, a measure of the effect of stress on body temperature [22,25], but had no significant impact on stress-coping behaviour in the forced swim test (FST) and the tail suspension test (TST) [22,28,42] (see summary in Table 1). In line with these NPS’s effects, the central infusion of NPS did not influence the genetically mediated stress-coping strategies in an animal model of depression-like behaviour (the Flinders Sensitive Line (FSL) rats [28]). In addition, no major effects of the NPSR1 deficiency were found on neuroendocrine and behavioural stress responsiveness in mice. For example, NPSR −/− and +/+ mice did not show significant differences in the basal CORT level [37,38], stress-induced CORT response (i.e., response to fear conditioning, forced swim testing or methamphetamine challenge) [30,38], stress-induced hyperthermia [26] and stress-coping behaviour in the FST and TST [26,30,52] (see summary in Table 2). 

### 2.4. Role of the NPS/NPSR1 System in Animal Models of Psychiatric Disorders

#### 2.4.1. The NPS/NPSR1 System and Pathological Anxiety and Fear

The administration of NPS or NPSR1 antagonists can reverse genetically-mediated pathological phenotypes in rodents. For example, NPS could reduce the innate high anxiety-related and aggressive behaviours of male mice and rats that are selectively bred for high anxiety-related behaviour (referred to as HAB) [31,48,53], which are considered as a genetic animal model of anxiety [54,55]. NPS also reduced the cued fear expression and facilitated the cued fear extinction in male HAB mice [31]. The anxiolytic-like effects of NPS in HAB mice were not related to changes in locomotion [31,53] and are associated with significant increases in *Nps* mRNA in the locus coeruleus and decreases in *Npsr1* mRNA in the BLA and PVN [31]. 

In both male mice and rats that show low anxiety-related behavior (LAB) and hyper-active stress-coping style, central NPS reduced the innate aggressive behaviour while the NPSR1 antagonist (d-Cys(tBu)5)NPS increased their low anxiety-related behaviour [31,48]. The anxiogenic and anti-aggressive effects of NPS were not reproduced by the infusion of NPS into the nucleus accumbens or the lateral hypothalamus (LH) [48], warranting further study of the neuroanatomical correlates by which the NPS/NPSR1 reverses the genetically-mediated pathological phenotypes in the HAB/LAB mouse lines.

The administration of NPS can also reverse genetically-mediated fear learning and memory deficits in mice. By using the genetically stress-susceptible 129S1/SvImJ mice showing, among other phenotypes, severe fear deficits (i.e., persistent defensive behaviour after fear extinction, enhanced fear generalization and deficits in safety learning) [56], Sartori and colleagues (2016) showed that central NPS infusion increased contextual discrimination and facilitated the contextual fear extinction in male 129S1/SvImJ mice. The effects of NPS on fear learning and memory were observed in the short-term as the central administration of NPS reduced freezing behaviour during extinction recall when mice were tested 3 days but not 13 days after the fear conditioning [57]. 

#### 2.4.2. The NPS/NPSR1 System and the Exposure to Acute Stressors

The administration of NPS can also reverse emotional behaviours produced by exposure to acute stressors. For example, the central infusion of NPS could reduce the anxiety-related behaviour of male mice subjected to social fear conditioning, which is a paradigm producing social avoidance of conspecifics [34]. It could also increase the sociability of male mice exposed to a 30 min social defeat session, considered as a relatively severe stressor based on social hierarchy and dominance [34]. In mice subjected to a 2 h immobilization stress, the intra-LA infusion of NPS could reduce the anxiety-related behaviour, facilitate the extinction of cued fear memory and reduce the contextual fear renewal [58]. The effects of NPS on fear memory were associated with reduced excitatory synaptic activity in the LA [58] and could be counteracted by the administration of the NPSR1 antagonist SHA68 [58]. 

The administration of NPS can also rescue PTSD-relevant alterations in fear-related behaviours. For example, the infusion of NPS into the ventromedial HYP or intra-BLA could reduce the anxiety-related behaviour of rats exposed to a single prolonged stress paradigm (i.e., 2 h of restraint stress followed by 20 min of FST) or a predator scent stress (PSS), respectively [43,51]. Both procedures produce alterations reminiscent of PTSD [59,60]. The PSS exposure also reduced the magnitude of their acoustic startle response and their freezing response to the re-exposure to the trauma cue (i.e., predator scent) [51]. Such effects were associated with increased brain-derived neurotrophic factor (BDNF) and neuropeptide Y1 receptor immunoreactivity in the dorsal hippocampus [51], suggesting significant changes in synaptic plasticity. 

Thus, parallel to the work on naive rodents, animal models based on stress exposure appear useful for investigating the mechanisms by which the NPS/NPSR1 system modulates emotional behaviours as well as how these mechanisms could contribute to the pathogenesis of stress-related disorders.

**Table 1:** Summary of the preclinical studies showing the role of the NPS/NPSR1 system in stress-relevant behaviours and responses.

Drug	Site	Test	Behaviour	Findings	Ref.
NPS	i.c.v.	EP/ZM, DaLi, MBT, DBT	Anxiety	↓ ♂/♀	[1,21,22,23,24,25,26,27,28,29,31]
		EPM, ETM		↔	[32,33,34]
	LA, BLA	EPM		↔	[51,58]
	LA/BLA	EPM, DaLi		↓	[41]
	MeA, VMH, PVN	EPM		↓	[21,40,43]
	VH	EPM		↓	[44]
		DaLi		↔	
	intranasal	EPM		↓	[23,53]
NPSR1-A	i.p. (SHA68)	EPM	Anxiety	↔	[43]
			Anxiolytic effects of intra-VMH NPS	X	
NPS	i.c.v.	FST, TST	Stress-coping	↔ ♂/♀	[22,28]
NPS	i.c.v.	SPAT	Sociability	↔	[23,34,48]
			Social recognition	↔	[23]
		RI	Aggression	↓ ↔	[48,49]
	intranasal	SPAT	Sociability	↔	[23]
NPSR1-A	i.c.v. ((d-Cys(tBu)5)NPS)	SPAT	Sociability	↔	[23]
	i.p. (SHA68)	RI	Anti-aggressive effects of i.c.v. NPS	Ø	[49]
	i.c.v. ((tBu-D-Gly5)NPS)		Anti-aggressive effects of i.c.v. NPS	X	
NPS	i.c.v.	CuFC	EXT	↑	[34]
			EXT recall	↔	
	LA	CuFC	EXP, EXT	↔	[58]
			Contextual fear renewal	↔	
	BLA	ASR	Magnitude	↔	[51]
		trauma cue	Freezing	↔	[51]
	LA/BLA	CuFC	EXP	↔	[41]
			EXT	↑	
			Contextual fear renewal	↓	
			EXT recall	↔	
NPSR1-A	i.c.v. ((d-Cys(tBu)5)NPS)	SFC	EXP	↔	[34]
	LA (SHA68)	CuFC	EXP, EXT	↔	[58]
			Contextual fear renewal	↔	
	LA/BLA (SHA68)	CuFC	EXT	↓	[41]
			Contextual fear renewal	↑	

All studies were conducted in male mice and rats, unless indicated in the table. Abbreviations: ASR, acoustic startle response; BLA, basolateral amygdala; CuFC, cued fear conditioning; DaLi, dark-light box test; DBT, defensive burying test; EPM, elevated plus-maze test; EP/ZM, elevated plus/zero-maze test; ETM, elevated T-maze test; EXP, expression; EXT, extinction; FST, forced swim test; i.c.v., intracerebroventricular; i.p., intraperitoneal; LA, lateral amygdala; MeA, medial amygdala; MBT, marble burying test; NPS, neuropeptide S; NPSR1-A, neuropeptide S receptor 1 antagonist; PVN, paraventricular nucleus of the hypothalamus; RI, resident intruder test; SFC, social fear conditioning; SPAT, social preference/avoidance test; TST, tail suspension test; VH, ventral hippocampus; VMH, ventromedial hypothalamus. Symbols: ↑, significant increase; ↓, significant decrease; ↔, no significant change; X, blocks NPS-induced effect; Ø, unable to block NPS-induced effects; ♂, male; ♀, female.

**Table 2:** Summary of the behavioural phenotypes observed in male and female mice with targeted disruption of the NPSR1.

Strain Background	Sex	Test	Behaviour	Findings	Ref.
C57/BL6J mice	♂/♀	EPM, DaLi, MBT	Anxiety	↔	[30,36,37,38]
		FST	Passive stress-coping	↑ ♂	[30]
		TST		↔	
		SPAT	Sociability	↔	[39]
			Social novelty	↔	
		SFC	EXP	↔	[39]
			EXT	↑	
		CFC	EXP	↔	[37]
			Context discrimination	↔	[37,38]
				↑	[37]
		CuFC	EXP, EXT	↔	[36]
		Safety learning	Freezing	↔	[38]
		ASR	Magnitude, Vmax	↔	[38,41]
				↓♂	[30,36]
		-	Basal CORT	↔	[37,38]
			Stress-induced CORT	↔	[30,38]
		Locomotion	Cocaine-induced	↔	[36]
CD1 mice	♂	EPM, ETM	Anxiety	↔	[26,33]
		FST	Stress-coping	↔	[26]
		RI	Aggression	↑	[49]
		SIH	Stress response	↔	[36]

Abbreviations: ASR, acoustic startle response; CFC, contextual fear conditioning; CORT, corticosterone; CuFC, cued fear conditioning; DaLi, dark-light box test; EPM, elevated plus-maze test; ETM, elevated T-maze test; EXP, expression; EXT, extinction; FST, forced swim test; MBT, marble burying test; RI, resident intruder test; SFC, social fear conditioning; SIH, stress-induced hyperthermia; SPAT, social preference/avoidance test; TST, tail suspension test. Symbols: ↑, significant increase; ↓, significant decrease; ↔, no significant change; ♂, male; ♀, female.

## 3. Role of the NPS/NPSR1 System in Addiction-Like Behaviours

The role of the NPS/NPSR1 system in addiction-like behaviours in rodents is summarized in Table 3. 

### 3.1. Role of the NPS/NPSR1 System in Drug-Induced Conditioned Place Preference

The role of the NPS/NPSR1 system in drug-induced conditioned place preference (CPP), which is a form of Pavlovian conditioning used to measure the rewarding or aversive effects of abused drugs, has been evaluated in male mice and rats (see summary in Table 3). In one study, NPS significantly reduced the expression of morphine-induced CPP but it did not affect the hyper-locomotor effects of morphine [61], suggesting distinct mechanisms for NPS modulation of the reinforcing and locomotor properties of this drug. In another study, NPS did not affect cocaine-induced CPP expression and extinction [62]. However, NPS increased the reinstatement of cocaine-induced CPP, which was blocked by the NPSR1 antagonist SHA68, an orexin receptor 1 (ORX1) antagonist (e.g., SB334867) or a cannabinoid receptor 1 (CB1R) antagonist (e.g., AM251). Such findings suggest that the reinstatement of cocaine-induced CPP by NPS likely involves NPS-cannabinoid-orexin cross-talks in brain areas involved in the behavioural motivation circuit [63]. This was further confirmed at the molecular level by the observation that NPS increased the activation of orexinergic neurons in the LH as well as enhanced orexin A levels in the ventral tegmental area (VTA) [62]. 

While NPS modulates the rewarding properties of morphine and cocaine, it seems to have no significant rewarding properties per se. Indeed, the central infusion of NPS in male rats produced a CPP when administered at the dose of 1 nmol and a conditioned place aversion at the dose of 0.01 nmol [64], whereas it had no rewarding or aversive properties in male mice [61,65]. Importantly NPS significantly increased the locomotor activity and the number of rearing at both doses producing CPP or CPA, yet without inducing locomotor sensitization [64]. Thus, similar to previous observations [61], the possible effects of NPS in the CPP paradigm were dissociable from the locomotor-activating effects of NPS.

### 3.2. Role of the NPS/NPSR1 System in Drug Self-Administration

#### 3.2.1. The NPS/NPSR1 System and Operant Self-Administration

The role of the NPS/NPSR1 system on drug-seeking behaviour using the operant intravenous (i.v.) paradigm has been investigated in male mice and rats (with the exception of one study) (see summary in Table 3). The NPS/NPSR1 system appears to have a major role in relapse to cocaine-seeking behaviour. Indeed, NPS significantly increased the cue-induced reinstatement of cocaine-seeking behaviour [24,66] whereas NPSR1 antagonists reduced the i.v. self-administration of cocaine and the cue-induced reinstatement of cocaine-seeking behaviour [66,67,68]. 

It is important to mention that NPSR1 antagonists reduced both the i.v. self-administration of cocaine and the self-administration of food when administered at the highest dose in both food restricted rats [68] and non-fasted rats [67], indicating that NPSR1 antagonists can reduce responses to drugs of abuse at low doses while they produce rather nonspecific effects on reinforcement behaviour at higher doses. Further work is required in order to understand the impact of the NPS/NPSR1 system on the regulation of food intake and eating behaviour (see for review [15]) and how this could interfere with other behavioural readouts. For example, NPS significantly reduced the palatable food intake in male rats [69] (see summary in Table 3) but the anorectic effects of NPS were observed at 30 min post-NPS infusion, possibly indicating a confounding effect of NPS-induced hyper-locomotion [69]. 

The AMY and the HYP appear to be important brain areas mediating the effects of NPS on the relapse of cocaine-seeking behaviour via NPSR1. This was indicated by the increased number of Fos-positive cells in these brain areas following the central infusion of NPS [66]. In addition, NPS and the NPSR1 antagonist (d-Cys(tBu)5)NPS directly infused into the LH or the perifornical area increased and decreased, respectively, the cue-induced reinstatement of cocaine-seeking behaviour in rats [66].

Similar to the observations on the reinstatement of cocaine-induced CPP, the effects of NPS on the reinstatement of cocaine-seeking behaviour seem to involve the orexinergic system [66]. They also appear to implicate the CRH system. This was indicated, on one hand, by the blockade of the reinstatement of cocaine-seeking behaviour by the subcutaneous injection of a CRHR1 antagonist (e.g., antalarmin) and, on the other hand, by the observation that NPS induced the reinstatement of cocaine-seeking behaviour in CRHR1 wild type (WT) mice but failed in CRHR1 KO mice [24]. Further studies are, however, required in order to understand the precise molecular mechanisms by which NPS interacts with the orexinergic and CRH systems to influence addiction-like behaviours.

While NPS modulates the reinforcing properties of cocaine, it seems to also possess such properties. Indeed, NPS was successfully centrally self-administered in male rats in range of doses that did not alter the locomotor activity [64]. The self-administration of NPS was blocked by a dopaminergic receptor D1 antagonist (e.g., SCH23390) or the ORX1 receptor antagonist SB33487 [64], suggesting that the reinforcing properties of NPS likely involve dopaminergic and orexinergic neurotransmissions.

#### 3.2.2. The NPS/NPSR1 System and Oral Self-Administration

The central infusion of NPS significantly reduced the intake of 20% ethanol in a voluntary limited access paradigm and the self-administration of ethanol in naïve rodents [65], as well as in genetically selected Marchigian Sardinian alcohol-preferring (msP) rats [32,70] (see summary in Table 3). While NPS failed to alter the cue-induced reinstatement of ethanol-seeking behaviour, the infusion of NPS into the LH was efficient in increasing this behaviour, which could be blocked by the administration of the ORX1 antagonist SB334867 [70]. Thus, the ethanol-seeking behaviour reinstated by NPS likely involves NPS-orexin cross-talks in the HYP. Interestingly, NPS reduced the intake of 15% ethanol in a two-bottle choice paradigm in female P rats (alcohol preferring) but not in female NP rats (non-alcohol preferring) [71]. Although such findings need to be reproduced, these results suggest that the effects of NPS on ethanol intake might be more readily revealed in female rodents displaying dysregulated behavioural phenotypes, which is a sex-specific response that deserves further investigation. Paradoxical to the above-described observations that NPS reduced ethanol intake [32,65,70], the NPSR1 antagonist NCGC84 reduced the intake and motivation for a 10% ethanol solution in the self-administration paradigm [72], highlighting that the NPS/NPSR1 system has a complex impact on the reinforcing properties of ethanol.

### 3.3. Role of the NPS/NPSR1 System in Drug Intoxication and Withdrawal

The NPS/NPSR1 system can modulate emotional behaviours produced by drug intoxication (see summary in Table 3). For example, mice given limited access to 20% ethanol showed increased anxiety-related behaviour, which was rescued by the central infusion of NPS [65]. Similarly, NPS reduced the anxiety-related behaviour in alcohol-preferring msP male rats [32]. The NPS/NPSR1 system can also rescue anxiety-related behaviours arising during drug withdrawal. For example, the central infusion of NPS reduced the anxiety-related behaviour produced by forced intra-gastric ethanol intoxication [73] or naloxone-precipitated morphine withdrawal [74]. These anxiolytic-like effects of NPS were accompanied by significant changes in *Npsr1* mRNA in several brain areas, including the BLA, bed nucleus of stria terminalis (BNST), PVN and VTA [73,74].

**Table 3:** Summary of the preclinical studies showing the role of the NPS/NPSR1 system in addiction-like behaviours.

Drug	Site	Test	Drug of Abuse	Behaviour	Findings	Ref.
NPS	i.c.v.	CPP	Cocaine	EXP, EXT	↔	[62]
				RST	↑	
			Morphine	EXP	↓	[61]
			NPS	EXP	↔	[61,65]
					↑	[64]
NPSR1-A	i.p. (SHA68)	CPP	Cocaine	RST	↔	[62]
				RST (stress)	X	
				RST (i.c.v. NPS)	X	
NPS	i.c.v.	oral	Ethanol	Intake	↓	[65]
				Anxiety	↓	
				Stress-coping	↓	
	BLA			Anxiety	↓	
	i.c.v.	IG	Ethanol	Anxiety (WD)	↓	[73]
		s.c.	Morphine	Anxiety (WD)	↓	[74]
NPS	i.c.v., LH, PeF	SA	Cocaine	SA (FR)	↔	[66]
				RST (cue)	↑	[24,66]
	DMH, CeA			RST (cue)	↔	[66]
	i.c.v.	SA	Ethanol	SA (FR)	↔	[32,70]
				RST (cue, NPS)	↑	
		TBC		Intake	↔ ♀	[71]
	LH	SA		RST (cue)	↑	[70]
NPS	i.c.v., LH, PVN	-	Palatable food	Intake	↓	[69]
	CeA			Intake	↔	
	i.c.v.	SA	NPS	SA (FR)	↑	[64]
NPSR1-A	i.p. (SHA68)	SA	Cocaine	SA (FR)	↔	[66]
					↓	[68]
				RST (cue)	↓	[66]
	i.p. (NPSR1-QA1)			SA (FR)	↔	[67]
				RST (cue)	↓	
	i.p. (RTI-118)			SA (FR)	↓	[68]
				RST (cue, stress, cocaine)	↓	
	i.c.v., CeA ((d-Cys(tBu)5)NPS)			RST (cue)	↔	[67]
	LH, PeF ((d-Cys(tBu)5)NPS)			RST (cue)	↓	[66,67]
	i.p. (NCGC84)		Ethanol	SA (FR, PR)	↓	[72]
				Motivation	↓	
				RST (cue)	↔	
	i.c.v. ((d-Cys(tBu)5)NPS)	-	Palatable food	Intake	↔	[69]
				Anorectic effects of i.c.v. NPS	X	

All studies were conducted in male mice and rats (except [71]). Abbreviations: BLA, basolateral amygdala; CeA, central amygdala; CPP, conditioned place preference; DMH, dorsomedial hypothalamus; EXP, expression; EXT, extinction; FR, fixed ratio; i.c.v., intracerebroventricular; IG, intragastric; i.p., intraperitoneal; LH, lateral hypothalamus; NPS, neuropeptide S; NPSR1-A, neuropeptide S receptor 1 antagonist; PeF, perifornical area; PR, progressive ratio; PVN, paraventricular nucleus of the hypothalamus; RST, reinstatement; SA, self-administration; s.c., subcutaneous; TBC, two-bottle choice test; WD, withdrawal. Symbols: ↑, increase; ↓, decrease; ↔, no significant change; X, blocks NPS-induced effect; ♂, male; ♀, female.

## 4. Alterations in the NPS/NPSR1 System in Stress-Related Disorders

Some behaviours modulated by the NPS/NPSR1 system (as reviewed here in Section 2 and Section 3) are affected in some stress-related disorders (e.g., PTSD, MDD and anxiety disorders) [19]. In addition, a potential association of the NPS/NPSR1 system with pathophysiological features of stress-related disorders and substance use disorder (SUD) has recently been proposed (see for review [18,20]). This association has especially been emphasized since the discovery of the SNP rs324981 in the human NPSR1 gene located on the chromosome 7p14.3. This SNP results in the change of an amino acid at position 107 (substitution Asn107Ile), which has marked functional consequences (i.e., ten-fold higher potency of NPS at the T allele (107Ile) encoded NPSR1 (Reinscheid, Xu, Okamura, et al., 2005).

### 4.1. Role of the NPS/NPSR1 System in Affective Disorders

A genotype-dependent association between the T allele and the risk for some anxiety and trauma-related disorders has been highlighted by several genetic association studies. The pioneering study of Okamura and colleagues found a reduced frequency of the AA genotype in adult Japanese men suffering from panic disorder (PD) [75]. In line with this, increased sensitivity to PD was observed in adult T-allele carrier Caucasian patients diagnosed with PD or another anxiety disorder (i.e., generalized anxiety disorder (GAD), social phobia, agoraphobia or phobia not otherwise specified) [76]. Similar to observations in PD, a trend for an underrepresentation of the AA genotype was observed in adult Asian patients with GAD compared to healthy controls [77]. In a recent study, an increased risk in developing PTSD following the Balkan war was revealed in carriers of the TT genotype [78]. Since the study of He and colleagues observed that the chronic administration of venlafaxine, a serotonin and norepinephrine reuptake inhibitor antidepressant, produced a greater reduction in anxiety symptom severity in GAD patients carriers of the AA or TT genotype compared to those with the AT genotype, further investigation of the impact of the SNP rs324981 on anxiety disease risk and response to pharmacological treatment is definitively required. Importantly, the plasma NPS level was found increased in adult Caucasian patients with GAD but not in patients with MDD compared to healthy controls, and the NPS level was positively related to the severity of anxiety symptoms [79,80], thus, providing evidence for a role of NPS/NPSR1 system in only a subset of affective disorders.

### 4.2. Role of the NPS/NPSR1 System in Substance Use Disorders

Complex interactions between the SNP rs324981, sex and increased alcohol use and subsequent alcohol use disorder (AUD) were found in Caucasian individuals [81]. In men, T-allele carriers showed increased alcohol use during late adolescence (before the age of 18) and increased risk for AUD in young adulthood (age of 25). Such vulnerability may have been brought about by experiences of adversity during adolescence (i.e., low warmth environment or stressful life events) or changes in personality traits following adversity (i.e., enhanced impulsivity and attention deficit hyperactivity disorder-related traits) [81]. In women, the A allele appeared as a vulnerability allele for both adolescent alcohol use and AUD in adulthood, which were associated with changes in personality traits after adversity, in particular, with increased maladaptive impulsivity and higher level of neuroticism [81]. The increased neuroticism in A-allele carrier women might be related to increased risk for affective disorders rather than AUD [82,83]. Surprisingly, no significant differences in genotype distribution were found in adult Japanese patients diagnosed with schizophrenia [75], which is a pathology characterized by high level of neuroticism [84], indicating that the relationship between the SNP rs324981, personality traits and risk for mental illness is very complex.

### 4.3. Role of the NPS/NPSR1 System in Anxiety Symptom Severity

A genotype-dependent association was suggested between the SNP rs324981 and anxiety symptom severity, although this association seems to depend on the primary medical condition. For example, a significant increase in anxiety symptom severity across psychiatric disorders was found in adult A-allele carrier Caucasian patients [85]. Increased frequency of the T allele was found in adult Caucasian hemodialysis patients with moderate or severe anxiety compared to those with low anxiety [86], whereas no significant differences in genotype distribution were observed in adult Chinese asthmatic patients with anxiety symptoms [87]. Similarly to the absence of changes in NPS level in patients with MDD, no significant differences in genotype distribution were observed in both hemodialysis and asthmatic patients with moderate or severe vs. low depression [86,87], thus confirming that the NPS/NPSR1 system is related to anxiety rather than depression. It is important to mention, however, that a significant increased depression score was found in asthmatic patients carriers of both the AA genotype for the NPSR1 gene and the GG genotype for the BDNF gene [87], indicating a synergistic interaction between BDNF and NPSR1 polymorphisms on depression symptoms. 

## 5. Role of the NPS/NPSR1 System in Emotion Regulation and Stress Responses in Healthy Individuals

The SNP rs324981 has a significant impact on emotion, stress regulation and brain activation correlates in healthy individuals (see summary in Table 4).

### 5.1. SNP rs324981, Anxiety Sensitivity and Fear Rating

A genotype-dependent association was found between the SNP rs324981 and anxiety sensitivity and fear rating (see summary in Table 4). In a fear conditioning paradigm using virtual reality contexts, individuals carrying the AA genotype showed increased anxiety rating of an anxiety-associated context during acquisition of fear conditioning as well as deficits in fear extinction, whereas T allele carrier individuals showed fear generalization [88]. In a cued fear conditioning paradigm, increased fear rating was observed in T allele carriers during the acquisition of fear conditioning [89]. In line with these observations, the SNP rs324981 had a major influence on startle magnitude to negative and neutral but not positive stimuli [88,90]. In particular, carriers of both the T allele for the NPSR1 gene and S allele for the serotonin-transporter-linked polymorphic (5-HTTLPR) region showed increased fear-potentiated startle magnitude [88], similar to observations of enhanced subcortical arousal in individuals diagnosed with various anxiety disorders [91,92,93]. It is important to mention that Domschke and colleagues (2012) found decreased startle magnitudes relative to unpleasant stimuli and increased startle magnitudes relative to neutral stimuli in T allele carrier individuals challenged with caffeine in an affect-modulated startle paradigm, which could reflect not only valence but also arousal effects. Further work is required in order to understand how differences in startle magnitude in otherwise healthy individuals may predict increased startle response relative to negative emotional stimuli in anxiety and anxiety disorders [94].

No significant effects of the SNP rs324981 on anxiety sensitivity were found in individuals presented with fearful/angry faces [95] or subjected to the emotional Stroop task [96], the emotional n-back test [97] or the emotional go/no go task [98]. In addition, no significant effect of the SNP rs324981 on anxiety sensitivity was observed in an attentional network task [99]. Furthermore, no genotype-dependent association was observed between the SNP rs324981 and depression or panic agoraphobia symptoms [95,96], as well as changes in maximum panic symptom scores during a pharmacological cholecystokinin tetrapeptide (CCK-4) panic challenge [100]. However, individuals carrying the TT genotype showed increased baseline panic symptom scores [100] as well as increased anxiety sensitivity if they experienced childhood trauma or recent life threatening events compared to individuals with the AA genotype [101]. Thus, the SNP rs324981 seems to have a minor impact on emotional symptoms of healthy individuals but interacts with other genetic polymorphism (e.g., 5-HTTLPR) or stress (e.g., childhood trauma or recent life threatening events) to modulate fear rating and anxiety sensitivity.

### 5.2. SNP rs324981, Coping Abilities and Experiences of Life Stress

A genotype-dependent association was found between the SNP rs324981, coping abilities and personality traits following adversity (see summary in Table 4). For example, women carrying the TT genotype and that experienced childhood maltreatment showed increased trait anxiety symptoms independently of general self-efficacy, which is a measure of coping abilities [102]. As for women carriers of the A allele that experienced childhood maltreatment, they showed increased anxiety symptoms only if they presented with low self-efficacy [102]. 

A genotype-dependent association was also found between the SNP rs324981 and some personality traits, which was modulated by adversity [103]. On one hand, young children to young adult carriers of the AA genotype showed increased adaptive impulsivity and increased openness to experience, whereas individuals carrying the TT genotype showed reduced adaptive impulsivity, reduced openness to experiences and increased hyperactivity [103]. Following the experience of stressful life events, individuals carrying the TT genotype showed increased adaptive and maladaptive impulsivity and increased hyperactivity; following a negative family environment, individuals carrying the AA genotype showed reduced adaptive and increased maladaptive impulsivity, increased neuroticism and reduced extraversion [103]. Thus, some personality traits present in individuals with the TT genotype may facilitate the development of anxiety or other symptoms independently of stress exposure.

### 5.3. SNP rs324981 and Neuroendocrine Stress Responsiveness

The SNP rs324981 has been found to have a minor impact on stress responses (see summary in Table 4). For example, when exposed to the trier social stress test (TSST), a procedure used to study stress hormone reactivity, T-allele carrier women showed reduced baseline CORT and T-allele carrier men showed increased baseline and stress-induced CORT when compared to individuals carrying the AA genotype [104,105]; however, the genotype differences did not reach statistical significance. In addition to the lack of significant effects of the SNP rs324981 on CORT levels in the TSST, no significant differences in stress-induced heart rate, salivary CORT and blood ACTH were found between genotypes in individuals exposed to the ScanSTRESS paradigm, a tool for stress induction in the magnetic resonance imaging environment [106] and during a pharmacological CCK-4 panic challenge [100]. In addition, the SNP rs324981 had no significant impact on subjective stress rating before and after the TSST [105] and no significant impact on skin conductance level in different emotional tasks [89,97].

**Table 4:** Summary of the clinical studies showing the impact of the SNP rs324981 on emotional symptoms, coping abilities/personality and stress responses in healthy individuals.

Parameter	TT	AT	AA	Ref.	
**Emotional symptoms**					
Anxiety sensitivity (emotional task)	↔	↔	↔//↑	[88]//[95,96,97,98]	
Anxiety sensitivity (attentional task)	↔	↔	↔	[99]	
Anxiety sensitivity (experience of life stress)	↑	↔	↔	[101]	
Panic agoraphobia	↔	↔	↔	[96]	
Baseline panic symptoms	↑	↔	↔	[100]	
CCK-4-induced panic symptoms	↔	↔	↔	[100]	
Depression symptoms	↔	↔	↔	[95,96]	
Fear rating (fear conditioning)	↑	↑	↔	[89]	
Startle magnitude to positive stimuli	↔	↔	↔	[90]	
Startle magnitude to negative stimuli	↓//↑$	↓//↑$	↔	[88]//[90]	
Startle magnitude to neutral stimuli	↑	↑	↔	[88,90]	
**Coping abilities and personality traits**					
Trait anxiety if low self-efficacy	↑	↑	↑	[102]	
Trait anxiety if high self-efficacy	↑	↓	↓		
Adaptive impulsivity	↓	↔	↑	[103]	
Adaptive impulsivity (experience of life stress)	↑	↔	↓		
Maladaptive impulsivity (experience of life stress)	↑	↔	↑		
Hyperactivity	↑	↔	↔		
Hyperactivity (experience of life stress)	↑	↔	↔		
Openness to experiences	↓	↔	↑		
Neuroticism (experience of life stress)	↔	↔	↑		
Extraversion (experience of life stress)	↔	↔	↓		
**Stress responses**					
Baseline CORT	↔	↔	↔	[104,105]	
↑ CORT (stress or CCK-4 challenge)	↔//↑♂	↔	↔	[100]//[104,105,106]	
↑ ACTH (stress or CCK-4 challenge)	↔	↔	↔	[106]	
↑ Heart rate (stress or CCK-4 challenge)	↔	↔	↔	[100,106]	
Skin conductance level (emotional task)	↔	↔	↔	[89,97]	
Subjective stress rating (TSST)	↔	↔	↔	[105]	

Clinical studies involved healthy individuals of both genders. The symbol ♂ refers to the effects only observed in men. The symbols TT, AT and AA corresponds to the three possible genotypes for the NPSR1 rs324981 single nucleotide polymorphism. Abbreviations: ACTH, adrenocorticotropic hormone; CORT, cortisol; CCK-4; cholecystokinin tetrapeptide; TSST, trier social stress test. Symbols: ↑, significant increase; ↓, significant decrease; ↔, no significant change; $, effects observed in carriers of the T allele for the NPSR1 gene and S allele for the serotonin-transporter-linked polymorphic region.

### 5.4. Neurobiological Mechanisms in Healthy Individuals 

The SNP rs324981 has been shown to modulate neural circuits following stress exposure or during emotional, cognitive or attentional tasks in healthy individuals (see summary in Table 5).

The SNP rs324981 was found to have a significant impact on the neural activation in a subset of brain areas involved in stress responses (anterior cingulate cortex (ACC)) or connected with stress-related brain areas (cerebellum and parahippocampal gyrus); however, findings differed by brain area and by gender. For example, increased stress-induced neuronal activation of the cerebellum was found in both men and women carriers of the TT genotype in the ScanSTRESS paradigm [106], while the stress-induced neuronal activation of the parahippocampal gyrus was significantly reduced and increased, respectively, in women and men with the TT genotype [104]. No significant changes in stress-induced neuronal (de)activation were found in the AMY and the ACC between genotypes [104,106]. By using proton magnetic resonance spectroscopy, increased metabolic activity of the ACC was revealed in carriers of the AA genotype but not in T-allele carriers during a pharmacological CCK-4 panic challenge [100]. 

The SNP rs324981 was also found to have a significant impact on the neural activation in various emotional tasks, particularly in the sub-regions of the prefrontal cortex (PFC) and the AMY. For example, in response to the presentation of negative, positive or neutral pictures in an emotional n-back test, increased, decreased or no change, respectively, were observed in the activation of the dorsolateral PFC (dlPFC) and the medial PFC (mPFC) in T-allele carrier individuals [97]. In addition, the anxiety sensitivity in T-allele carriers negatively correlated with the activation of the left dlPFC and mPFC to negative pictures. In other studies, T-allele carrier individuals showed increased activation of the AMY and increased (de-)activation of the middle frontal gyrus, hippocampus, insula and striatum to fearful/angry faces [95]. In a Westphal paradigm, they displayed increased activation of the AMY and a trend for a significant increase in the activation of the inferior orbitofrontal cortex when presented with agoraphobic pictures [107]. Finally, T-allele carriers also exhibited increased activation of the right rostral dorsal ACC/dorsomedial PFC to the conditioned stimulus during the acquisition of a cued fear conditioning [89]. Altogether, these results show that T-allele carrier individuals generally present with decreased activation of the PFC to positive stimuli and increased activation of different sub-regions of the PFC and the AMY to negative stimuli. Such patterns of brain activation suggest that the SNP rs324981 influences the neural response to emotional stimuli, which may facilitate the development of stress-related disorders.

The SNP rs324981 had a significant impact on the neural activation in cognitive and attentional tasks, although the number of studies is quite limited. In a cognitive Stroop task, increased activation of the dlPFC was revealed in individuals with the AA genotype compared to those with the TT genotype [96]. In this study, individuals carrying the AA genotype showed increased activation of both the dlPFC and the mPFC when presented with fearful stimuli [96]. This activation pattern was not observed in individuals carrying the TT genotype and was interpreted by the authors as a possibly increased inhibitory control of the PFC over the AMY. In an attentional task, individuals carrying the TT genotype differed to A-allele carriers in their activation of various brain areas (right superior parietal lobule, right PFC and locus coeruleus area) in the alerting condition (i.e., presentation of cue vs. no cue), as well as during the executive condition (i.e., congruent vs. not congruent) [99]. In addition, a significant positive correlation was found between the anxiety scores and the PFC activation in individuals carrying the TT genotype [99], which might be interpreted as a compensatory mechanism for increased bottom-up signals of the alerting system (i.e., increased activity of the locus coeruleus).

## 6. Conclusions

In the present review, we have provided a comprehensive overview about the current state of knowledge of the role of the NPS/NPSR1 system in emotionality, stress responsiveness and addiction-like behaviours in rodents. We have also described findings on the potential association of the NPS/NPSR1 system with pathophysiological features of stress-related disorders. 

As reviewed, there is substantial clinical and preclinical evidence for a role of the NPS/NPSR1 system in the behavioural and emotional changes relevant to stress-related disorders and substance abuse. However, much remains to be investigated, especially with respect to the cellular and molecular mechanisms by which the NPS/NPSR1 system is associated with stress responses and subsequent stress-related phenotypes relevant to mental health. An important point to consider is that the findings from the experimental rodent models highlight the effects of the administration of NPS and NPSR1 antagonists in various brain regions on stress responses and reward-like and emotional behaviours (see summary in Table 1 and Table 3), whereas the majority of the clinical studies focused on the impact of the SNP rs324981 on behavioural and emotional changes in healthy individuals (see summary in Table 4 and Table 5), and only a few studies involved patients with a psychiatric disorder. Thus, it appears that comparing the role of the NPS/NPSR1 system in both animal models and the human condition is very challenging.

Regarding behavioural changes relevant to substance abuse, studies in naïve rodents show that NPS generally reduces drug intake and CPP, as well as emotional behaviours produced by drug intoxication and withdrawal, but paradoxically increases relapse to drug-seeking behaviour (see summary in Table 3 and Figure 1). 

Only one clinical study investigated the impact of the SNP rs324981 on drug use and abuse and found that the T allele in men (i.e., associated with increased NPSR1 efficiency) and the A allele in women were more so associated with pathological drug use [81]. It is important to mention that only one study investigated the impact of NPS on reward-related behaviour in female rodents [71]. Since there is increasing evidence in humans and laboratory animals for biologically based sex differences in every phase of the drug addiction process (see for review [108]), further studies should investigate whether and how the NPS/NPSR1 system interacts with sex to influence addiction-like behaviours.

Regarding emotional behaviours and stress responses, studies in naïve rodents have shown that NPS generally increases locomotion and exploration, reduces anxiety-related and aggression-related behaviours, facilitates the extinction of fear memory and increases HPA axis activity (see summary in Table 1 and Figure 1). The behavioural phenotyping of male and female NPSR1 −/− mice showed only slight abnormalities in anxiety-related, aggression-related and fear-related behaviours (see summary in Table 2), probably reflecting the confounding effects of developmental compensation. 

As for substance abuse, very few studies investigated the impact of NPS on emotional behaviours and stress responses in female rodents despite the existing major sex differences in emotional behaviours in rodents and underlying mechanisms (for review see [109,110]). By contrast to findings in animals, clinical studies showed the AA genotype was associated with increased anxiety and the T allele with fear generalization in healthy humans, while no major genotype differences were found in anxiety sensitivity, HPA axis (re-)activity and stress rating in various tasks (see summary in Section 5). Thus, further studies are required in order to determine whether and how the NPS/NPSR1 system modulates addiction-relevant behaviours, emotional behaviours and stress responses under non-pathological conditions.

Importantly, a few rodent studies examined the effects of NPS and NPSR1 antagonists on emotional behaviours in rodents with innate pathological phenotypes (i.e., high or low anxiety-related behaviour, depression-like behaviour and fear deficits) or exposed to acute stress. These studies found that, similar to naïve rodents, the NPS/NPSR1 system not only regulates anxiety and fear memory but also social/aggressive behaviours (see summary in Section 2.4). Quite oppositely, clinical studies found that the T allele was associated with increased risk for stress-related disorders involving dysregulation in anxiety and arousal (e.g., GAD, PD and PTSD) and increased risk for anxiety symptoms in patients with a medical condition (see summary in Section 4). Thus, better translational approaches are crucially needed to further our understanding of the role of the NPS/NPSR1 system, especially the SNP rs324981, in mental health. Since the SNP has not been described in rodents that only present Ile at NPSR1 position 107 and its role is uncertain [111], Bengoetxea and colleagues have recently established transgenic mouse lines expressing the ancestral NPSR1-I107 variant or the human NPSR1-N107 variant [47]. Interestingly, mice carrying the hypo-functional N107 variant displayed an improved extinction of conditioned fear compared to mice carrying the ancestral I107 variant, with sex differences revealed when adjusting the salience of fear training [47]. Given the genetic complexity at the NPSR1 locus and the functional consequences of the NPSR1 rs324981 SNP, behavioural and emotional changes relevant to stress-related disorders are currently under further investigation using this “humanized” mouse model. 

Despite the above considerations, the studies reviewed and presented herein demonstrate that pharmacological interventions targeting the NPS/NPSR1 system display much promise for the treatment of numerous stress-related disorders. Indeed, NPS possesses anxiolytic-like properties in rodents that are similar to those of conventional anxiolytic drugs (e.g., diazepam, chlordiazepoxide and alprazolam) [22,25,27]. However, NPS promotes arousal, and its anxiolytic-like actions could not be blocked by flumazenil, a benzodiazepine antagonist [22], indicating that the anxiolytic-like effects of NPS are not mediated via benzodiazepine-like action on GABA receptor-chloride ionophore complex. Moreover, NPS reduced palatable food intake in male rats [69]. The anorectic effects were blocked by the central administration of the NPSR1 antagonist (d-Cys(tBu)(5))NPS but not by a CRHR antagonist (i.e., alpha-helical CRF 9–41) [69]. The anorectic effects of NPS, thus, contrast with the effects of conventional anxiolytic drugs such as benzodiazepines, which interfere with the HPA axis activity by antagonizing CRH and for which its anxiolytic properties are generally associated with increased food intake [112,113]. Despite numerous challenges inherent in developing therapeutics targeting neuropeptide systems (see for review [114]), future pharmacological studies should, thus, consider targeting the central NPS/NPSR1 system for treating stress-related disorders. 

Encouragingly, NPS delivered to the brain by intranasal infusion had beneficial effects on anxiety-related behaviour in rodents, which were likely achieved by influencing NPS-responsive systems in brain areas regulating emotion and stress responses. For example, intranasal NPS administration reduced anxiety-related behaviour in the elevated plus-maze test in male rats and mice, without significant impact on locomotion [23,53]. The anxiolytic effects of intranasal NPS were accompanied by reduced short-term and long-term plasticity in the ventral hippocampus [44,53], as well as alterations of some glutamatergic genes (i.e., increased expression of glutamate transporter 1 and synapsin II) in the PFC [53]. These studies show a clinically relevant and non-invasive therapeutic approach for the delivery of NPS to the brain [115]. Further studies are necessary in order to determine if intranasal NPS can reverse other impairments relevant to stress-related disorders. It is important, however, to mention that the intranasal NPS application seemed to possess a relatively long period of time in terms of the onset of effects (i.e., the anxiolytic-like effects were observed at 4 h but not 30 min post-NPS infusion [53]). In addition, it was associated with reduced NPS uptake in many brain areas when compared with central NPS administration (e.g., cortex, AMY, locus coeruleus, basal ganglia, cerebellum and Barrington’s nucleus) [53]. Nevertheless, the reviewed findings illustrate the important role of NPS in regulating emotion and stress responses relevant to stress-related disorders, and the findings point to its potential as a therapeutic agent that can be administered intranasal.

## Figures and Tables

**Table 5:** Summary of the clinical studies showing the impact of the SNP rs324981 on neural activation in various tasks in healthy individuals.

Brain Area	TT	AT	AA	Ref.
**Neural activation to stress or CCK-4 challenge**				
Parahippocampal gyrus	↑♂, ↓♀	↔	↔	[104]
Amygdala	↔	↔	↔	[104,105]
Anterior cingulate cortex	↔	↔	↔//↑	[100]//[104,106]
Cerebellum	↑	↔	↔	[106]
**Neural activation in an emotional task**				
Dorsolateral prefrontal cortex				
Positive stimuli	↓	↓	↔	[96,97]
Negative stimuli	↑	↑	↑	
Neutral stimuli	↔	↔	↔	
Medial prefrontal cortex				
Positive stimuli	↓	↓	↔	[96,97]
Negative stimuli	↑	↑	↑	
Neutral stimuli	↔	↔	↔	
Rostro dorsal anterior cingulate cortex/Dorsomedial prefrontal cortex	↑	↑	↔	[89]
Amygdala	↑	↑	↔	[95]
**Neural activation in a cognitive task**				
Dorsolateral prefrontal cortex	↔	↔	↑	[96]
Medial prefrontal cortex	↔	↔	↔	
**Neural activation in an attentional task**				
Superior parietal lobule	↑	↔	↔	[99]
(Dorsolateral) Prefrontal cortex	↑	↔	↔	
Locus coeruleus	↑	↔	↔	

Clinical studies involved healthy individuals of both genders. Abbreviations: CCK-4; cholecystokinin tetrapeptide. Symbols: ↑, significant increase; ↓, significant decrease; ↔, no significant change.

## Data Availability

Data sharing not applicable.
